# Problems associated with the use of the term “antibiotics”

**DOI:** 10.1007/s00210-021-02144-9

**Published:** 2021-09-18

**Authors:** Roland Seifert, Bastian Schirmer

**Affiliations:** Institute of Pharmacology, Hannover Medical School, Carl-Neuberg-Str. 1, 30625 Hannover, Germany

**Keywords:** Antibiotics, Antibiogram, Antibiotic stewardship, Broad-spectrum antibiotics, Reserve antibiotics, Chemotherapeutics

## Abstract

The term “antibiotics” is a broadly used misnomer to designate antibacterial drugs. In a recent article, we have proposed to replace, e.g., the term “antibiotics” by “antibacterial drugs”, “antibiosis” by “antibacterial therapy”, “antibiogram” by “antibacteriogram”, and “antibiotic stewardship” by “antibacterial stewardship” (Seifert and Schirmer Trends Microbiol, 2021). In the present article, we show that many traditional terms related to antibiotics are used much more widely in the biomedical literature than the respective scientifically precise terms. This practice should be stopped. Moreover, we provide arguments to end the use of other broadly used terms in the biomedical literature such as “narrow-spectrum antibiotics” and “reserve antibiotics”, “chemotherapeutics”, and “tuberculostatics”. Finally, we provide several examples showing that antibacterial drugs are used for non-antibacterial indications and that some non-antibacterial drugs are used for antibacterial indications now. Thus, the increasing importance of drug repurposing renders it important to drop short designations of drug classes such as “antibiotics”. Rather, the term “drug” should be explicitly used, facilitating the inclusion of newly emerging indications such as antipsychotic and anti-inflammatory. This article is part of an effort to implement a new rational nomenclature of drug classes across the entire field of pharmacology.

**Supplementary Information:**

The online version contains supplementary material available at 10.1007/s00210-021-02144-9.

## Why the term “antibiotic” is problematic

Literally, the term “antibiotic” means “directed against life”. However, in reality, antibiotics designate drugs directed against bacteria. But, in fact, antibiotics are still both prescribed by professionals and expected by patients for treatment of non-bacterial diseases, most notably virus-caused diseases such as acute respiratory infections (Dhingra et al. 2020; Ray et al. 2019). The broad use of antibiotics in general medicine against “flu-like” diseases of the upper respiratory tract is a particularly bad example for overprescription of antibiotics (Fleming-Dutra et al. 2016; McDonagh et al. 2018). This misuse of antibiotics is a consequence of numerous interconnected factors, including misconceptions about the indication of antibiotics, intercollegial dynamics, patient expectations, and normative beliefs (Akkerman et al. 2005; Faure et al. 2009; Warreman et al. 2019). It can be hypothesized that the use of the historic misnomer “antibiotic” contributes to each of these factors. Because the socio-cultural/psycho-social research on the determinants of antibiotic resistance development is a quite young discipline, further research is needed to firmly establish such a contribution of imprecise terminology (Donisi et al. 2019). Regardless of the causes of misuse of antibacterial drugs, their inappropriate use has resulted in the emergence of numerous resistant bacterial strains including multi-resistant (or methicillin-resistant) *Staphylococcus aureus* (MRSA). These bacterial strains cause serious hospital-acquired infections and have led to the closure of whole wards (Curtis et al. 2019; Wu et al. 2019; Oliver et al. 2020; Zhao et al. 2020). Knowledge-based and psycho-social intervention programs targeting prescribers, “consumers”, and pharmacists have proven beneficial in reducing misuse of antibacterial drugs (Altiner et al. 2007; Burstein et al. 2019; Hickman et al. 2003). The use of precise terms in such interventions might help to clearly and unequivocally define the problems and challenges of antibacterial stewardship and prevention of emerging resistances.

Based on this linguistically and medically inacceptable situation, we have recently proposed to replace the term “antibiotics” by the term “antibacterial drugs” (Seifert and Schirmer 2021). This proposed change in nomenclature is actually consistent with the well-established use of the terms “bacteriostatic drugs” and “bactericidal drugs”. Related to this change in nomenclature, the term “antibiosis” should be replaced by “antibacterial therapy”, “antibiogram” by “antibacteriogram”, “antimicrobial” by “antipathogenic drug”, “antibiotic agents” by “antibacterial drugs”, “antibiotic activity” by “antibacterial potency”, and “antibiotic stewardship” by “antibacterial stewardship”. Table 1 provides the definitions of some important pharmacological terms in the field of antibacterial and antipathogenic therapy.

**Table 1:** Definitions of some important pharmacological terms in the field of antibacterial and antipathogenic therapy

Pharmacological term	Definition
Antibacterial drug	A drug that possesses inhibitory effects on bacteria. Bacteriostatic drugs inhibit the growth of bacteria without killing them. Bactericidal drugs kill bacteria
Antibacterial resistance	The uncritical use of antibacterial drugs in medicine and agriculture has resulted in ineffectiveness of many antibacterial drugs in important diseases
Antibacterial stewardship	This constitutes an interdisciplinary approach of microbiologists, pharmacologists, pharmacists, and clinicians to optimize the use of antibacterial drugs in patient treatment and to avoid development of antibacterial resistance
Antibacteriogram	In an antibacteriogram, a pathogenic bacterium is cultured and the minimum inhibitory concentration (MIC) of various antibacterial drugs on bacterial growth is assessed. The antibacteriogram provides a rational basis for selecting the best antibacterial drug for a patient
Antimycotic (antifungal) drug	A drug that possesses inhibitory effects on fungi. Mycostatic (fungistatic) drugs inhibit the growth of fungi. Mycocidal (fungicidal) drugs kill fungi
Antiparasitic drug	A drug that possesses inhibitory effects on parasites. Parasites include protozoa, worms, and ectoparasites
Antipathogenic drug	An umbrella term for drugs including antibacterial drugs, antimycotic drugs, antiparasitic drugs, and antiviral drugs
Antiviral drug	A drug that possesses inhibitory effects on viruses. Virtually all antiviral drugs are virustatic; i.e., they interfere with the reproduction of viruses in human cells
Bactericidal drug	A drug that kills bacteria. Prototypical bactericidal drugs are penicillins, cephalosporins, fluoroquinolones, and aminoglycosides
Bacteriostatic drug	A drug that inhibits the growth of bacteria without killing them. Prototypical bacteriostatic drugs are tetracyclines, macrolides, and lincosamides
Chemotherapeutic	Historic umbrella term for antipathogenic drugs (antibacterial drugs) and cytostatic drugs
Drug	A drug is a chemical substance with beneficial effects on human health. In contrast, poisons possess detrimental effects on human health
Drug repurposing	This procedure describes a strategy of using already approved drugs for new clinical indications beyond the traditional uses. The advantage of drug repurposing is that it is much less expensive than the de novo development of drugs because important parameters such as pharmacokinetics, drug interactions, and adverse effects are already known
MIC (minimum inhibitory concentration)	This is the lowest effective concentration of an antibacterial drug at which it shows an inhibitory effect on bacterial growth in an antibacteriogram
MRSA (multidrug (methicillin)-resistant *Staphylococcus aureus*)	Strains of *Staphylococcus aureus* that are resistant to multiple antibacterial drugs including methicillin. The uncritical use of antibacterial drugs is a major driver of MRSA development
Mycobactericidal drug	A drug that kills mycobacteria. Isoniazide (INH), rifampicin (RMP), and pyrazinamide (PZA) are prototypical mycobactericidal drugs
Mycobacteriostatic drug	A drug that inhibits the growth of mycobacteria without killing them. Ethambutol (EMB) is a prototypical mycobacteriostatic drug
Potency	Potency defines the concentration at which a drug exhibits 50% of its maximum effect. Many antipathogenic drugs are enzyme inhibitors. Therefore, the potency of many antipathogenic drugs refers to the inhibitory drug concentration causing 50% of enzyme inhibition (IC_50_)

## Problematic traditional terms are deeply rooted in the biomedical literature

**Fig. 1:**
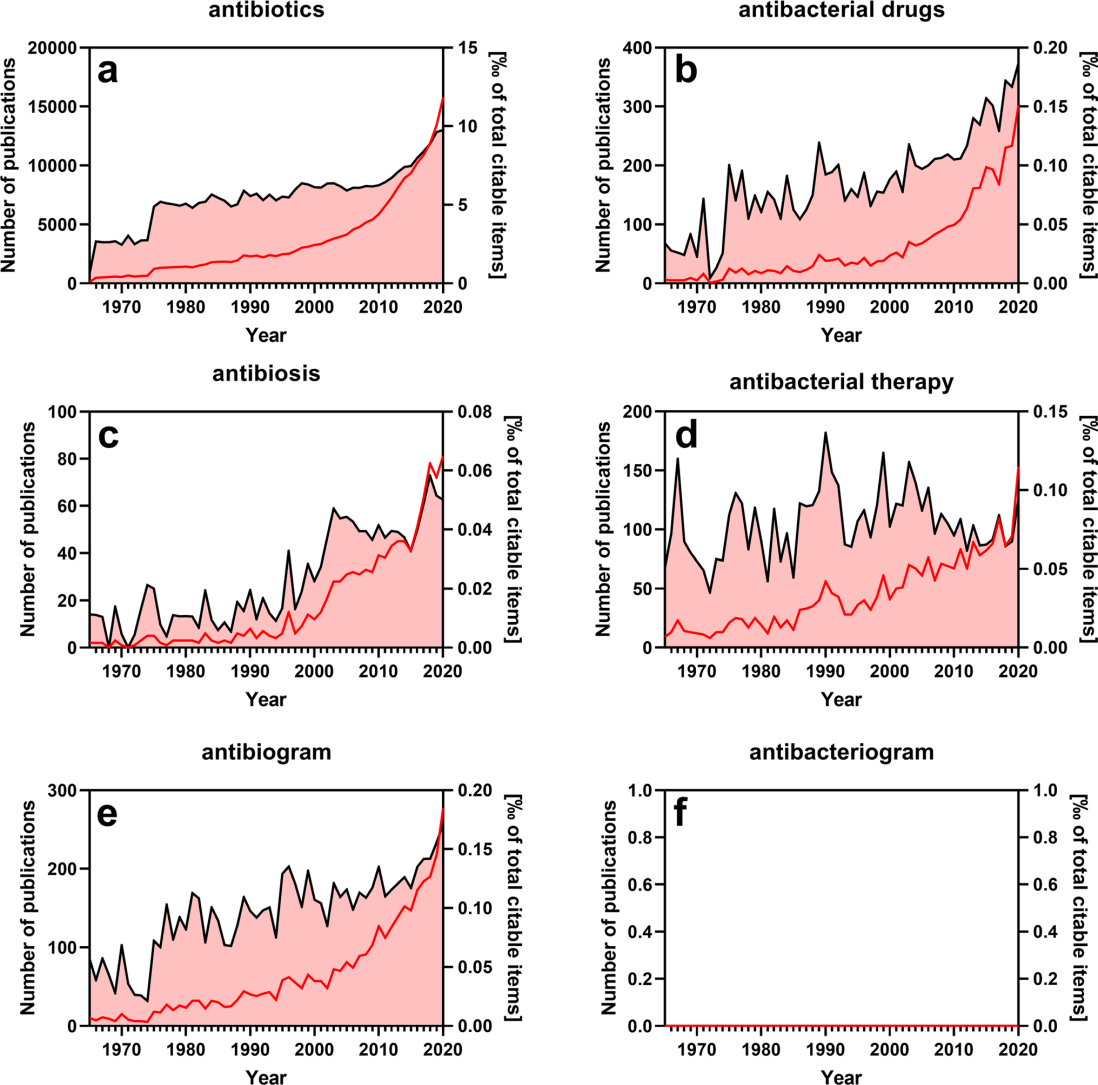

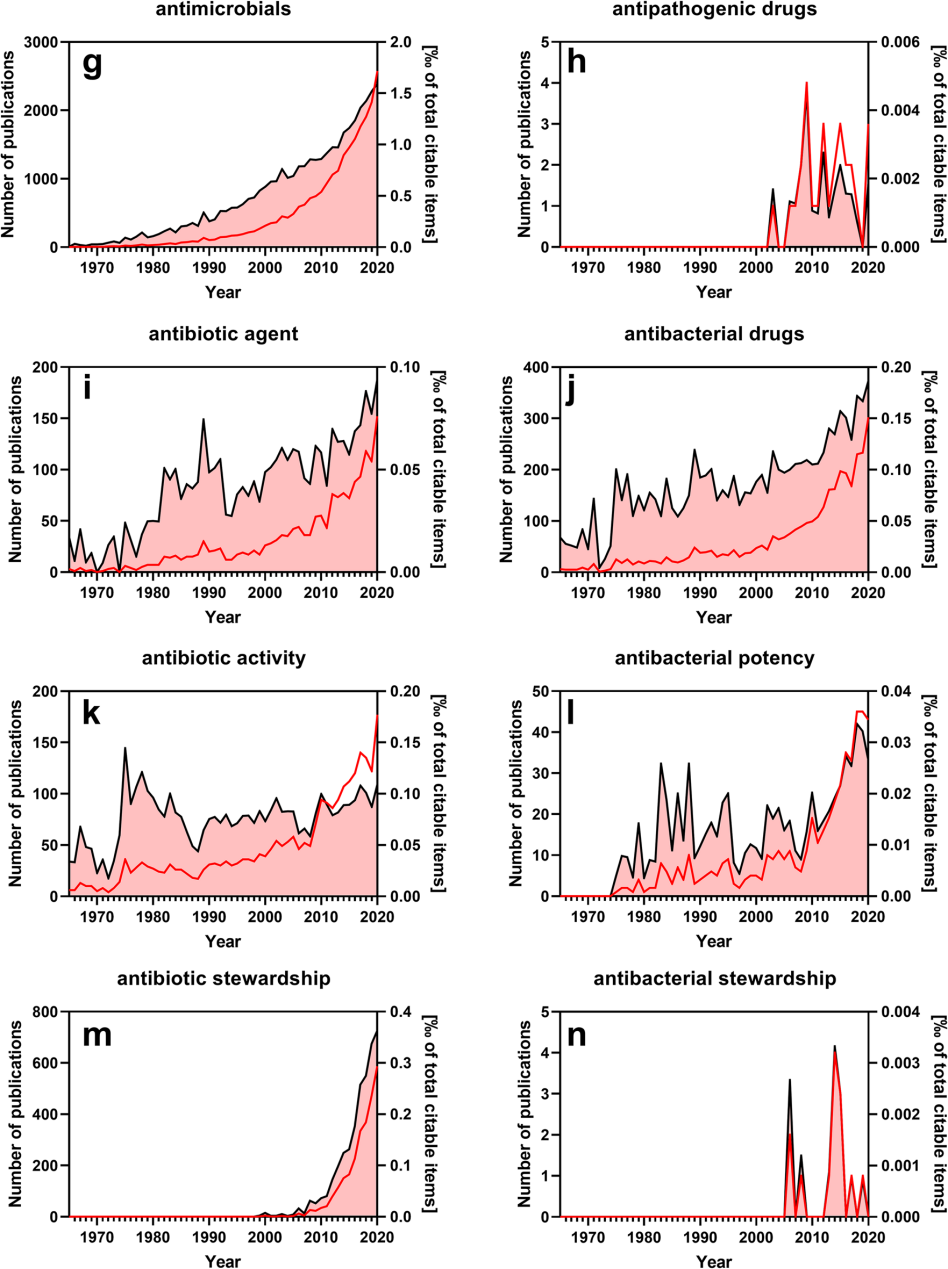
Citation frequency of modern and traditional terms related to antipathogenic drugs. The PubMed search was performed on May 13, 2021, and was confined to titles and abstracts of searchable items. Depicted in the plots are the absolute number of publications per year that use a specific term (red line/left *y*-axis) and the relative number of these publications normalized to the total number of citable items of the corresponding year (black line/right *y*-axis). Except for panels **a**, **g**, **m**, and **n**, both singular and plural forms of the search term have been included

To assess how often traditional terms are used in the biomedical literature, we analyzed scientific articles indexed in PubMed and compared the citation frequency of traditional (imprecise) terms versus that of scientifically precise terms (Fig. 1). It is quite astonishing that the imprecise term “antibiotics” is used 50 times more often than the precise term “antibacterial drug” (compare Fig. 1a versus Fig. 1b). This discrepancy is an indication that convention rather than critical reflection determines the use of this term in scientific literature. However, the imprecise term “antibiosis” and the precise term “antibacterial therapy” (compare Fig. 1c and d) are used with similar frequency which is inconsistent to the comparisons shown in Fig. 1a and b. Thus, as previously discussed for the terms “bactericidal” and “bacteriostatic”, precise terms are not necessarily ignored in the literature. Most strikingly, the imprecise term “antibiogram” (derived from “antibiotic”) is very broadly used in the literature, even with increasing frequency, whereas the precise term “antibacteriogram” was not found in a single PubMed-indexed publication under our search conditions at all (Fig. 1e versus Fig. 1f). In fact, even a non-confined Google search with the term “antibacteriogram” currently yields only ~ 60 hits (search date May 14, 2021).

The diffusely defined term “antimicrobials” is also excessively used in the literature, while the precise term “antipathogenic drugs” is rather uncommon (compare Fig. 1g and h). The historic term “agent”, which is poorly defined pharmacologically, is also deeply rooted in the biomedical literature although the precise term “antibacterial drug” has become more prevalent in recent years (compare Fig. 1i and j).

As stated recently, the term “activity” should be reserved to receptor agonists possessing intrinsic activity and stabilizing a pharmacologically active receptor conformation. Most antibiotic “agents” are actually enzyme inhibitors and not receptor agonists. Nonetheless, the imprecise term “antibiotic activity” is used far more commonly in the literature than the term “antibacterial potency” (compare Fig.1k and l).

As a result of improper and unreflective use of antibacterial drugs and the emergence of bacterial resistances, the biomedical field of antibacterial stewardship was developed to improve the rational use of these drugs in the clinics and fight development of bacterial resistances (Ha et al. 2019). Ironically, these important measures to improve proper use of antibacterial drugs have not yet penetrated scientific language. The imprecise term “antibiotic stewardship” is used more than 100-fold more often than the precise term “antibacterial stewardship” (compare Fig. 1m and n). Taken together, these examples illustrate that imprecise language use in the field of antibacterial therapy is very common in the biomedical literature. This is, however, not a trivial issue because language also strongly influences and shapes thinking and decision processes (Mahon and Kemmerer 2020). In this regard, there is clearly much to be done in the scientific language.

It could be argued that abandoning traditional terms would render literature searches more difficult. We concur that for a transition period, this may be the case, but in the long run, there is no alternative to using the precise term because the dissociation between traditional terminology and actual scientific meaning has become too large over the past seven to eight decades. In the future, the gap between traditional terms and their precision will inevitably further increase. The increased length of the precise terms is a possible downside of our proposed nomenclature, and this may be suspected to complicate communication. But based on the practical experience of the authors using the adapted nomenclature in scientific articles, teaching texts, and lectures, this is rarely the case.

## More problematic terms linger in the literature

**Fig. 2:**
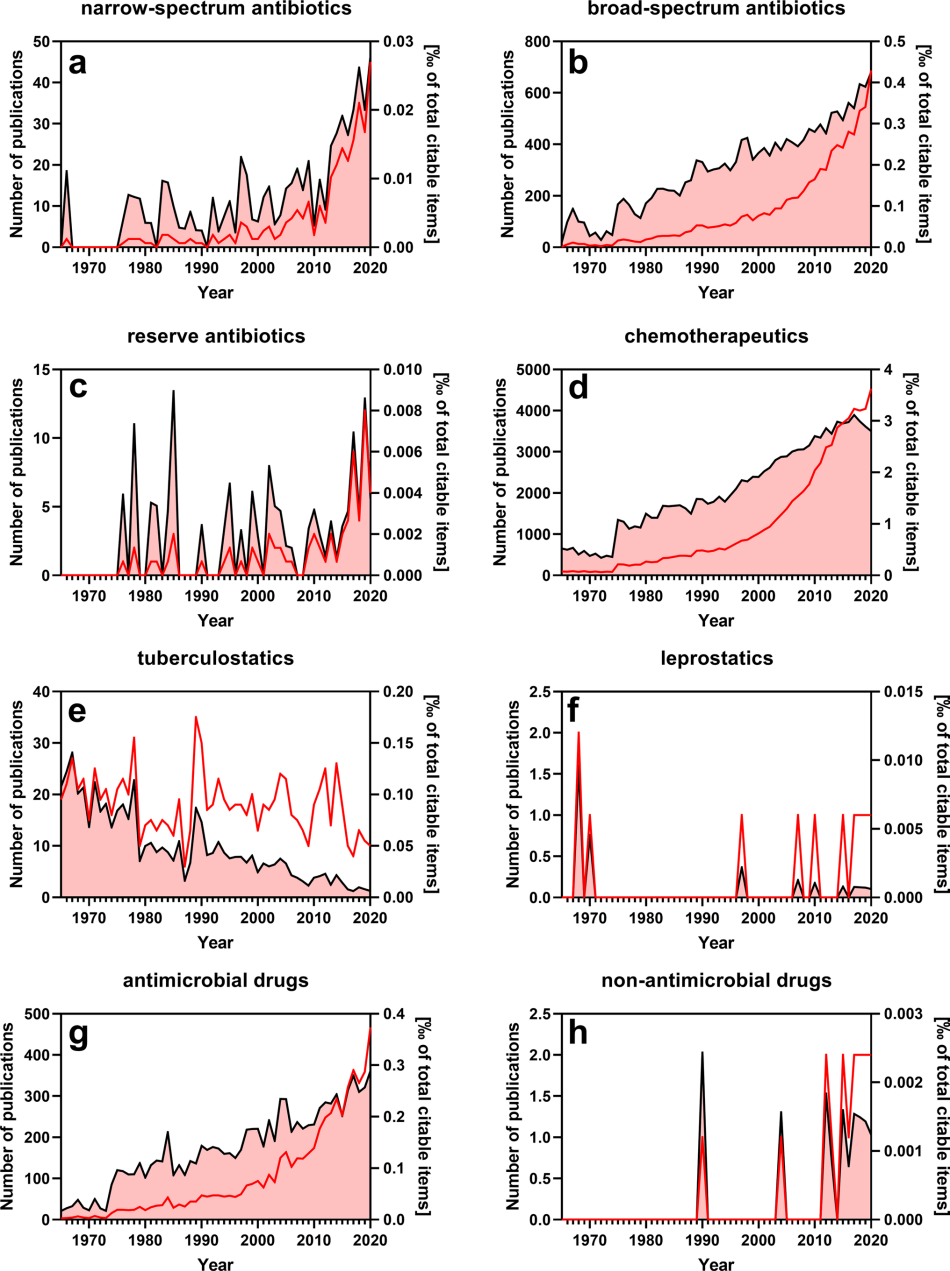
Citation frequency of several traditional terms related to antibacterial drugs that should not be used anymore. The PubMed search was performed on May 13, 2021, and was confined to titles and abstracts of searchable items. Depicted in the plots are the absolute number of publications per year that use a specific term (red line/left *y*-axis) and the relative number of these publications normalized to the total number of citable items of the corresponding year (black line/right y-axis). Both singular and plural, hyphenated and non-hyphenated forms of the respective search term have been included

Table 2 lists several problematic terms in the field of antibacterial therapy that should not be used anymore and provides reasons for avoiding them. Figure 2 illustrates the use of these problematic terms in the biomedical literature. One of the most widely used problematic terms is “broad-spectrum antibiotics”. In fact, with increasing resistance of bacteria against broad-spectrum antibiotics (antibacterial drugs), the term “broad-spectrum antibiotics” is used with increasing frequency (Fig.2b) as if increasing the use of an imprecise term would alleviate the medical problem. Unfortunately, the term “broad-spectrum antibiotics” is not precisely defined. Quite different definitions exist which drugs are included in this definition. In fact, the definition of the term varies greatly with respect to time and geographical location, reflecting the dynamic resistance situation of pathogenic bacteria (Curtis et al. 2019; Wu et al. 2019). The term “narrow-spectrum antibiotics” is similarly poorly defined as the “broad-spectrum antibiotics” but also used with increasing frequency in the biomedical literature. In fact, due to the uncritical use of broad-spectrum antibiotics, several of these drugs have been converted to narrow-spectrum antibiotics meanwhile (Karam et al. 2016; Vivas et al. 2019). Related to the latter two terms, the term “reserve antibiotics” is used in the literature (Fig.2c), but due to their uncritical use, many reserve antibiotics have lost this status now, leaving us empty-handed without therapeutic alternatives (Remschmidt et al. 2017; Richter et al. 2019; Annamalai et al. 2021).

**Table 2:** Traditional terms related to antibacterial drugs that should not be used anymore

Traditional term	Reason why the traditional term should not be used anymore	Reference for use
Antimicrobial drugs	The term is too broad in the sense that it refers all types of “microbes”. In fact, in medicine, we are interested only in interfering with pathogenic microorganisms. Microorganisms belonging to the microbiome have beneficial effects and should not be adversely affected by antipathogenic drugs. Hence, the term antipathogenic drugs is more precise	(Sokol et al. 2007; Mulder et al. 2020)
Non-antimicrobial drugs	This is a classic negative definition of a drug class with no common underlying mechanism. An analogous term is “non-opioid analgesics” encompassing various mechanistically diverse drugs. The term “non-antimicrobial drugs” is defined far too imprecisely because every drug that originally has no antimicrobial effect is included in this class. However, since nowadays several so-called non-antimicrobial drugs are being repurposed for treatment of diseases caused by pathogens, the former term causes only confusion and should be dropped	(Pereira et al. 2018)
Broad-spectrum antibiotics	There is no generally accepted definition which antibacterial drug is a broad-spectrum antibacterial drug. In fact, the spectrum of pathogenic bacteria covered by a given antibacterial drug varies greatly in terms of time and geographical location. Due to uncritical use the “spectrum” of many antibacterial drugs has become narrower during the past years. The term “broad spectrum” also conveys the false impression to the physician that all or at least most pathogenic bacteria are coved by a broad-spectrum antibacterial drug. But in contrast, this misconception increases the probability that resistances emerge	(Gerber et al. 2017; Curtis et al. 2019; Wu et al. 2019; Joyner et al. 2020)
Narrow-spectrum antibiotics	In fact, as the result of the uncritical use of “broad-spectrum” antibacterial drugs, several of these drugs have become “narrow-spectrum” antibacterial drugs. Thus, like the term “broad-spectrum”, the term “narrow-spectrum” is not clearly defined. Dropping these two misleading terms honestly acknowledges the fact that the spectrum of antibacterial drugs changes temporarily and geographically	(Gerber et al. 2017; Curtis et al. 2019; Wu et al. 2019; Joyner et al. 2020)
Reserve antibiotics	Originally, the use of reserve antibacterial drugs was restricted to cases in which “broad-spectrum” and “narrow-spectrum” antibacterial drugs did not work anymore. However, the increasing resistance problem has resulted in an expansion of the use of “reserve” antibacterial drugs beyond the originally intended indications into traditional fields of “broad-spectrum” and “narrow-spectrum” antibacterial drugs. Hence, like for the other types of antibacterial drugs, the term “reserve antibacterial drugs” lacks a clear definition. Rather, a given antibacterial drug must be assigned to a specific pathogenic bacterial strain and a clearly defined clinical use	(Robertson et al. 2019)
Chemotherapeutics (chemotherapeutic agents)	Historic term used to designate antipathogenic drugs or antibacterial drugs. Sometimes, the term “chemotherapeutics” designates only synthetic antibacterial drugs, but often natural (e.g., fungus- or plant-derived) antibacterial drugs are included as well. To complicate matters, the term “chemotherapeutics” also includes classic cytostatic drugs used for the treatment of malignant tumors. In current language, the term “chemotherapy” is almost exclusively used for therapy of malignant tumors, but not for pathogen-caused diseases. The term “chemo” also has a negative connotation, signaling harm, danger and toxic effects. This should be avoided because several antipathogenic drugs are tolerated very well. Moreover, the term “chemotherapeutics” also alludes to the existence of allegedly “good” biotherapeutics, but the term “biotherapeutics” is uncommon in medicine. Instead, the term “biologicals” is broadly used, also suggesting via the prefix “bio” that these drugs have few if any adverse effects	(Nandi et al. 2020; Layeghi-Ghalehsoukhteh et al. 2020)
Leprostatics	The term “leprostatics” is used as an umbrella term to cover both leprostatic and “leprocidal drugs”. Because it is important to discriminate between the two classes of drugs, the incorrect umbrella term should be dropped. The term “anti-leprosy drugs” is a more appropriate umbrella term. The term “antimycobacterial drugs” also covers anti-tuberculosis drugs	(Caliskan et al. 2019)
Tuberculostatics	The term “tuberculostatics” is often used as an umbrella term to cover both tuberculostatic and tuberculocidal drugs. Isoniazide, pyrazinamide and rifampicin are prototypical tuberculocidal drugs, while ethambutol is a tuberculostatic drug. Because it is important to discriminate between the two classes of drugs, the incorrect umbrella term should be dropped. The term antimycobacterial drugs also covers anti-leprosy drugs	(Damasceno Junior et al. 2020)

One of the most widely used terms in the field of antibacterial therapy is “chemotherapeutics”, and its use in the biomedical literature increases (Fig. 1d). Again, the term lacks an unequivocal definition. Historically, the term was initially coined to describe chemically synthesized antibacterial drugs (as opposed to naturally occurring antibiotics). Later, both natural and chemically synthesized antibacterial drugs were included in this category. Subsequently, the chemically synthesized cytostatic drugs with anti-tumor effects were included in this broad category as well. However, nowadays, in the general medical communication and in the media (TV, radio, Internet), the term chemotherapy is almost exclusively used to describe an anti-tumor therapy with cytostatic drugs. Moreover, the syllable “chemo” has a negative connotation of “bad” and “many side effects” (adverse drug reactions, ADR) as opposed to the syllable “bio”, implicating beneficial effects without harmful side effects (Wakiuchi et al. 2019; Ihrig et al. 2020). However, these associations are completely wrong.

Another popular term in the biomedical literature is “tuberculostatics” (Fig. 2e). This term alludes to the fact that these drugs inhibit the growth of *Mycobacterium tuberculosis*. However, in the biomedical literature, this term is also incorrectly used to include tuberculocidal drugs (Damasceno Junior et al. 2020; García-Caballero et al. 2020). Therefore, the precise terms “tuberculostatic drugs” and “tuberculocidal drugs” should be used. Similar considerations apply to the infrequently used term “leprostatics” including both “leprostatic drugs” and “leprocidal drugs” (Caliskan et al. 2019).

Like the term “chemotherapeutics”, the term “antimicrobial drugs” is not clearly defined, but nevertheless widely used in the literature. In fact, with the advent of drug repurposing, i.e., the use of old drugs for new therapeutic purposes, several traditional antibacterial (antipathogenic) drugs are now used for indications unrelated to bacterial diseases (Table 3). Conversely, many drugs traditionally used for the treatment of diseases unrelated to pathogen-caused diseases are now being repurposed for the treatment of pathogen-caused diseases (Table 4). This development has resulted in the term “non-antimicrobial drugs”. However, this term is a negative definition without a common mechanism of action or chemical structure. Therefore, the term “non-antimicrobial drugs” should not be used anymore, like the “non-opioid analgesics”, also including mechanistically heterogenous classes of drugs with diverse mechanisms of action. The nomenclature problem becomes even more evident considering the traditional and non-traditional designations of drug classes in the context of new indications (Tables 3 and 4).

**Table 3:** Repurposing of antipathogenic drugs for indications beyond pathogen-caused diseases

Antipathogenic drug or drug class	Traditional indication	New indication	Reference
Chloroquin (antimalarials, disease-modifying antirheumatic drugs, DMARDs)	Malaria	Lupus erythematosus, rheumatoid arthritis	(Rainsford et al. 2015)
Doxycyclin, minocyclin (tetracyclines, antibiotics)	Bacterial infections, malaria	Schizophrenia, major depressive disorder, neurodegenerative diseases, cancer therapy	(Husain et al. 2017; Socias et al. 2018; Ben-Azu et al. 2018; Schmidtner et al. 2019; Antoszczak et al. 2020; Issy et al. 2020)
Erythromycin (macrolides, macrolide antibiotics, broad-spectrum antibiotics)	Bacterial infections	Gastric hypomotility	(Jun et al. 2014; Zimmermann et al. 2018)
Fluconazole (triazoles, azole antimycotics, antimycotics)	Fungal infections	Hypoxic pulmonary vasoconstriction	(El-Sherbeni and El-Kadi 2016; Kandhi et al. 2017)
Ivermectin (antiparasitics, antiparasitic drugs)	Parasitic diseases	Treatment of alcoholism, cancer therapy	(Yardley et al. 2015; Antoszczak et al. 2020)
Ceftriaxone (cephalosporins, antibiotics, β-lactam antibiotics, broad-spectrum antibiotics, broad-spectrum cephalosporins)	Bacterial infections	neurological disorders, drug dependency/withdrawal	(Yimer et al. 2019)
Rifampicin (ansamycins, tuberculostatics, tuberculocidal drugs, antimycobacterial drugs)	Mycobacterial infections	Neurodegenerative diseases	(Socias et al. 2018)
Ciclopirox	Fungal infections	Porphyria, ischemic stroke	(Urquiza et al. 2018; Feng et al. 2020)
Metronidazole (nitroimidazoles, antianaerobials, chemotherapeutics)	Bacterial infections	*Trypanosoma cruzii* infection	(Simões-Silva et al. 2017)
Macrolides (macrolide antibiotics, broad-spectrum antibiotics)	Bacterial infections	Chronic inflammatory diseases (asthma/chronic obstructive lung disease, osteoarthritis, cystic fibrosis), acute respiratory distress syndrome, sepsis, pneumonia	(Zimmermann et al. 2018; Reijnders et al. 2020)

In parentheses, representative names of drug classes (both traditional and mechanistic) are provided

**Table 4:** Repurposing for pathogen-caused diseases of drugs not traditionally used for pathogen-caused diseases

Drug or drug class	Traditional indication	New indication for pathogen-caused disease	Reference
Auranofin (disease-modifying antirheumatic drugs, DMARDs)	Rheumatoid arthritis	Inhibition of biofilm formation	(She et al. 2019; Jang and Eom 2020)
COX-inhibitors (e.g., diflunisal, piroxicam)	Osteoarthritis, rheumatoid arthritis	Infections with *S. aureus* and cryptococci	(Ogundeji et al. 2016; Carta et al. 2018)
Dihydropyridines (e.g., nitrendipine)	Hypertension	*H. pylori* infection	(González et al. 2019)
5-Fluorouracil (classic cytostatics, pyrimidine analogs)	Various malignant tumor diseases	Bacterial infections	(Soo et al. 2016)
Metformin (biguanides, oral antidiabetics)	Type-2 diabetes	Malaria, trypanosomiasis, bacterial infections, hepatitis B	(Butts et al. 2014; Pryor and Cabreiro 2015; Honda et al. 2016; Kapoor et al. 2018; Martínez-Flórez et al. 2020)
Propranolol (β-adrenergic receptor antagonists, β_x_AR antagonists)	Migraine prophylaxis, tremor, infantile hemangioma	Hepatitis, *C. albicans*	(Ueno et al. 2009; Kapoor et al. 2018)
Thalidomide (hypnotics)	Sleeplessness in pregnant women	Tuberculous meningitis, leprosy	(Walker et al. 2015; Kumar et al. 2020)
Disulfiram	Treatment of alcoholism	*Borrelia burgdorferi* infection	(Potula et al. 2020)
Tamoxifen (selective estrogen receptor modulators, SERM)	Breast cancer	Cryptococcal infections	(Butts et al. 2014; Hai et al. 2019)
Statins (HMG-CoA reductase inhibitors, e.g., atorvastatin, fluvastatin)	Hypercholesterinemia	Fungal infections	(Macreadie et al. 2006)
Sertraline (selective serotonin reuptake inhibitors, SSRI)	Major depressive disorder	*S. aureus* biofilm formation, fungal infections, parasitic infections	(Weeks et al. 2018; Muthu et al. 2019; Gowri et al. 2020)
Lorazepam (benzodiazepines, allosteric GABA_A_-receptor modulators)	Sedation, anxiety disorders	Fungal infections	(Kathwate et al. 2015)
Ticagrelor (irreversible P2Y_12_-receptor antagonists)	Thrombosis prevention and therapy	*C. difficile* infection	(Phanchana et al. 2020)

In parentheses, representative names of drug classes (both traditional and mechanistic) are provided

These few examples highlight that the field of antibacterial (antipathogenic) drug therapy is abound with highly problematic terms that may deteriorate the precision of drug therapy, cause confusion, render literature searches increasingly difficult, and hinder patient communication. The eminent presence of problematic terms in the biomedical literature reflects the fact that authors, journal editors, and peer reviewers alike are not yet sufficiently aware of the issue and/or do not act accordingly.

## First simple steps to improve terminology

Many traditional terms in the field of (antibacterial) drug therapy have become highly problematic. Particularly problematic is the use of the common prefix “anti” followed by a brief description of the drug class such as “antibiotics”. A simple immediate solution to the problem is to rigorously avoid all problematic terms, even if they have a long history, and rather use precise terms that do not bear the risk of confusion. As the most important example, instead of using the term “antibiotics”, the term “antibacterial drugs” should be used. The apposition of the simple word “drug” to a given drug class renders the term more precise, because this opens up the possibility that an antibacterial drug also possesses, e.g., antidepressive or antipsychotic effects (example of tetracyclines, Table 3). Conversely, the use of the term “anti-inflammatory drug” also leaves open the possibility that this drug additionally exhibits antibacterial effects (example of diflunisal and piroxicam, Table 4).

The process of abandoning the traditional drug nomenclature, highlighted by the case of antibiotics, will not only be important for pharmacology textbooks, the biomedical literature, and professional communication, but also for physician–patient communication. How should a patient understand that the doctor is prescribing an antibiotic for depression or schizophrenia? As a first step towards solving the problems associated with the use of traditional drug nomenclature in the public, one of the authors of this article has recently published a book in German language for a general audience explaining these problems (Seifert 2021). As the next step, the book will be adapted into English language for an international general audience.

## What else needs to be done in the future

How will the revised nomenclature be viewed by international learned societies? Implementation and acceptance of the proposal will require endorsement by international learned societies. First of all, the drug nomenclature proposal will be discussed by the International Union of Basic and Clinical Pharmacology (IUPHAR). Similarly, the proposal must be discussed and approved by the International Union of Microbiological Societies (IUMS).

How will the nomenclature be integrated in textbooks and teaching? A textbook in English language has partially implemented some of the aspects discussed in this article (Seifert 2019). The textbook in German language (Roland Seifert, Basiswissen Pharmakologie (German language), second edition, Springer, 2021) has already fully implemented the proposed new nomenclature. The Federal Institute for Medical and Pharmaceutical Exam Questions (IMPP) in Germany has adopted the new nomenclature as well (https://www.impp.de/pruefungen/allgemein/gegenstandskataloge.html, accessed on May 14, 2021). Hence, the next generation of physicians in Germany will be familiar with the new nomenclature. The modern nomenclature facilitates learning, and students embrace the new nomenclature because of its logic. It will be more challenging for professors and lecturers to switch to the new nomenclature.

How will the nomenclature be implemented in the scientific literature? The traditional nomenclature renders literature searches extremely difficult and biased. The historic scientific track record cannot be changed anymore, but journal editors can gradually implement new nomenclature by amending the instructions for authors. But again, this change will require time because of the large number and heterogeneity of scientific journals.

How will the nomenclature be integrated into daily scientific communication? This issue probably represents the biggest hurdle because all scientists were socialized with the traditional nomenclature without even reflecting its problems.

## Summary

Resistance against antibacterial drugs has become a major problem because of uncritical use of these drugs. Drug repurposing in the field of antipathogenic drugs becomes more important. Antipathogenic drugs are used for indications beyond pathogen-caused diseases, and drugs traditionally used in other fields are increasingly used for pathogen-caused diseases. Traditional terminology in the field of antipathogenic drugs is becoming increasingly imprecise. Terms such as “antibiotics”, “antibiogram”, “agent”, and “activity” should be dropped. A precise drug nomenclature based on chemical and mechanistic considerations is proposed. Terms such as “antibacterial drugs”, “antibacteriogram”, “drug”, and “potency” should be used instead of traditional terms. We are convinced that a precise terminology will improve the precision of science, assist reducing drug resistance, and improving antibacterial stewardship. To our knowledge, studies investigating the association between using correct medical terms and correct drug use are missing so far. Further research is needed to provide definitive evidence for this impact of using correct terms.

## Supplementary Information

Below is the link to the electronic supplementary material.

Supplementary file1 (XLSX 77 KB)

## Data Availability

The raw data of the literature search is amended to the main article as supplementary material (MS Excel data file).
